# A service evaluation of passive remote monitoring technology for patients in a high-secure forensic psychiatric hospital: a qualitative study

**DOI:** 10.1186/s12888-023-05437-w

**Published:** 2023-12-14

**Authors:** Lindsay H. Dewa, Josephine Broyd, Rita Hira, Alison Dudley, Jonathan D. Hafferty, Robert Bates, Paul Aylin

**Affiliations:** 1 National Institute for Health Research Imperial Patient Safety Translational Research Centre, Institute of Global Health Innovation, Imperial College London, London, UK; 2 School of Public Health, Imperial College London, Reynolds Building, London, W6 8RP UK; 3 West London NHS Trust, London, UK

**Keywords:** Patient safety, Mental health, Inpatient, Forensic, Secure care, Digital mental health, Passive monitoring, Qualitative, Service evaluation

## Abstract

**Background:**

Technology has the potential to remotely monitor patient safety in real-time that helps staff and without disturbing the patient. However, staff and patients’ perspectives on using passive remote monitoring within an inpatient setting is lacking. The study aim was to explore stakeholders’ perspectives about using Oxehealth passive monitoring technology within a high-secure forensic psychiatric hospital in the UK as part of a wider mixed-methods service evaluation.

**Methods:**

Semi-structured interviews were conducted with staff and patients with experience of using Oxehealth technology face-to-face within a private room in Broadmoor Hospital. We applied thematic analysis to the data of each participant group separately. Themes and sub-themes were integrated, finalised, and presented in a thematic map. Design, management, and analysis was meaningfully informed by both staff and patients.

**Results:**

Twenty-four participants were interviewed (*n* = 12 staff, *n* = 12 patients). There were seven main themes: detecting deterioration and improving health and safety, “big brother syndrome”, privacy and dignity, knowledge and understanding, acceptance, barriers to use and practice issues and future changes needed. Oxehealth technology was considered acceptable to both staff and patients if the technology was used to detect deterioration and improve patient’s safety providing patient’s privacy was not invaded. However, overall acceptance was lower when knowledge and understanding of the technology and its camera was limited. Most patients could not understand why both physical checks through bedroom windows, *and* Oxehealth was needed to monitor patients, whilst staff felt Oxehealth should not replace physical checks of patients as reassures staff on patient safety.

**Conclusions:**

Oxehealth technology is considered viable and acceptable by most staff and patients but there is still some concern about its possible intrusive nature. However, more support and education for new patients and staff to better understand how Oxehealth works in the short- and long-term could be introduced to further improve acceptability. A feasibility study or pilot trial to compare the impact of Oxehealth with and without physical checks may be needed.

**Supplementary Information:**

The online version contains supplementary material available at 10.1186/s12888-023-05437-w.

## Background

High-secure forensic inpatient mental health services provide mental health care and treatment to improve mental health recovery and reduce risk to the public for patients considered at the gravest risk. Across the four UK high-secure hospitals, there are up to 800 patients at any one time. Forensic inpatients often exhibit interpersonal violence and aggression, and experience other safety incidents including falls and suicidal behaviour [1]. Staff manage these incidents before they occur using risk assessment and observations, and following the behaviour can respond with containing interventions (e.g., restraint, seclusion). Formal risk assessments (e.g. Historical Clinical Risk – 20 risk assessments, HCR-20) typically occur every 3 months, which limit detection of rapid momentary changes in behaviour, vital signs and physical health [2], and as most patient observations are through the door windows into the bedroom and every 15–30 min, incidents and health decline can be missed. Moreover, physical checks can be labour intensive and unsafe for staff. Moreover, the paradoxical effect of intermittent vital sign checks in-person can cause insomnia, and is linked to patient agitation, aggression, and suicidal behaviour [3, 4], which poses a threat to patient safety.

All patients in a high-secure hospital in the UK are detained under the Mental Health Act 1983 and subject to monitoring to ensure patient safety. Passive remote monitoring technology (e.g., non-invasive measures) has the potential to monitor patient safety continuously in real-time without disturbing or interacting with the patient. Oxevision (i.e. Oxehealth)^1^ is a non-invasive CE-marked medical device that supports staff to monitor patient activity remotely to improve patient safety. Oxehealth includes an infrared-sensitive camera that enables staff to visually confirm that the patient is safe and check their vital signs (i.e., pulse and breathing rate) periodically (15-s maximum spot check and stored for up 24 h as encrypted video images and then overwritten). It can also detect location-based alerts that are potentially high-risk (e.g., getting out of bed, patient spends an unusually long time in the bathroom) to help staff decide whether to intervene in real-time to prevent safety incidents [5].

Oxehealth was subject to rigorous consideration and assessment of data protection and privacy in line with legislation (e.g., GDPR, UK Data Protection Act 2018 and the European Convention on Human Rights – Article 8) and guidelines (i.e., Caldicott Principles on patient-identifiable data). Oxehealth Implementation was formally signed off by the Chief Medical Officer and Caldicott Guardian on behalf of each NHS Trust board. Potential risks to data protection and privacy invasion have been mitigated by ensuring strict protocols of the camera are implemented to ensure any un-pixelated video can only be viewed with express permission in exceptional circumstances (e.g., potential risk to the patient). Patients are informed about the use of Oxehealth upon admission including the use of the camera and monitoring however there are concerns that the system raises ethical concerns particularly on privacy, consent, and human rights. In contrast, there is also a need to protect patients and keep them safe. This ongoing debate, acknowledged within guidance [6], on the acceptability of Oxehealth requires further investigation.

Oxehealth has been implemented within several mental health hospitals across the UK, including Broadmoor Hospital in South England. Some initial feasibility work and peer reviewed evaluation has been conducted in these hospitals, where studies have found that Oxehealth can detect deterioration, improve patient safety [7] and is cost-saving [8]. However, most studies have been quantitative in design, have small sample sizes and/or from the staff perspective only. Qualitative research exploring experiences in high-secure forensic mental health services in general is limited, particularly in the patient population [9]. It is important to understand the end-user view of Oxehealth to see if it can be successfully integrated long-term. Qualitative evaluation is crucial to bring an enriched understanding and experiences of Oxehealth within a local context. Our study aim was to conduct a qualitative service evaluation to explore both staff and patient perspectives on the use of Oxehealth technology in a high-secure forensic psychiatric hospital.

## Methods

**Fig. 1:**
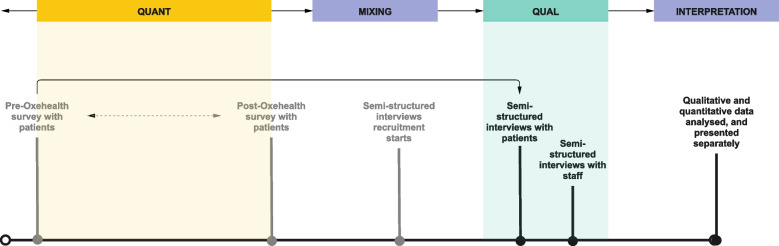
Explanatory sequential mixed methods process applied to service evaluation

This study was part of a larger explanatory sequential mixed methods service evaluation, but each phase has been reported separately. A survey (QUANT) was conducted first, and this was followed by semi-structured interviews (QUAL) (Fig. 1). The qualitative phase reported here was guided by the recommended consolidated criteria for reporting qualitative research (COREQ) [10] (Additional file 1). We took a phenomenological approach to understand the perspectives of staff and patients of the Oxehealth implementation.

### Participant selection and setting

A purposive sample of patients with mental disorders were recruited from Broadmoor Hospital in South England within West London NHS Trust. Patients were eligible to take part if they had completed at least one questionnaire from the quantitative phase (Dewa LH, Broyd J, Hira R, Hafferty J, Aylin P: A service evaluation of passive remote monitoring technology for patients in a high-secure forensic psychiatric hospital: a descriptive study, in preparation). Inclusion and exclusion criteria were the same as the quantitative phase but briefly noted here, patients were included if they had capacity to consent, fluency in English language, and could be interviewed with minimal risk of harm to staff. Staff participants were eligible to take part if they had experience of using Oxehealth technology in Broadmoor Hospital. Patient participants were approached in a randomised order from a list of all participants who had completed the quantitative phase (Dewa LH, Broyd J, Hira R, Hafferty J, Aylin P: A service evaluation of passive remote monitoring technology for patients in a high-secure forensic psychiatric hospital: a descriptive study, in preparation). Staff were approached via individual email in first instance, and reminder follow-up requests were administered after a week.

### Data generation

All participants who had completed the quantitative phase were entered into an excel spreadsheet, assigned an anonymised ID and randomised using the random number generator option. They were then approached between December 2022 and March 2023 by the researcher team (JB, RH) in the order on the list. If participants did not wish to take part, the next person on the list was approached. The researchers (JB, RH), who had established experience of working with high-secure forensic patients, conducted semi-structured interviews face-to-face in a private room. Information sheets were shared with each participant and informed written consent was obtained.

Two topic guides were utilised, one for patients and one for staff. The patient topic guide was co-produced with two patients with lived experience of high-security, Oxehealth and mental health difficulties and structured as follows: (1) patient understanding and awareness of Oxehealth technology; (2) impact of Oxehealth on wellbeing; (3) impact on quality of life; (4) impact on behaviour and safety; (5) impact for others including staff; (6) impact when in seclusion, and (7) future of Oxehealth. The staff topic guide was adapted based on the patient topic guide and informed by two staff members with lived experience of Oxehealth (AD, one other staff member). Two additional areas were included: staff usage of Oxehealth and different formats of using Oxehealth (e.g., computer, IPad). All interviews were audio recorded and then transcribed verbatim in-house within the hospital grounds by an independent administration staff member. Patient participants received £5 each as an incentive in line with hospital policy and in-house ethical guidelines.

### Patient and public involvement

Patient and public involvement (PPI) and specifically co-production in high-secure forensic mental health hospitals is limited. We used learning from our previous studies to adapt our approach and to suit the forensic psychiatric setting [11]. A group of patients that were deemed clinically stable and interested in research were approached to join the team as research partners. Two patients with lived experience (advisors) indicated they were interested in getting involved and joined the project team. We met face-to-face during a 2-h meeting before Covid-19 to introduce the project, get to know each other, ascertain expectations and goals going forward. Both advisors then reviewed and edited the information sheet, and consent form through an iterative approach between them, the researchers on site (JB, RH), and the lead researcher (LD). We adapted our approach to include face-to-face meetings where possible and on a 1–1 basis with the research assistants when face-to-face was not possible due to restrictions (e.g., Covid-19 lockdowns, no staff to escort/observe patients).

Due to these project delays (i.e., Covid-19 lockdowns) one advisor left the project, and another person joined the team. The researchers (JB, RH) met the advisors regularly (i.e. often weekly) in person on the ward to update them about the project progression, and how their feedback had informed documents and next steps. We had another face-to-face 2-h meeting in the hospital during Covid-19 to co-produce the topic guide for the patient interviews. We wrote up all potential topic areas of discussion and incoroporated them into a draft topic guide. We then sent the draft back to the two advisors for their approval and sign off. A similar process happened for the staff interview topic guide but occurred via the research assistant meetings with the patients. One patient advisor attended a 2-h face-to-face meeting with the research team (LD, JB, RH) to co-create the initial coding framework and initial themes. This was then shown to the other patient on a 1–1 basis whose feedback informed the next analysis stage. Both patient advisors were paid in accordance with the hospital pay guidance (£5 per interview). PPI was reported according to the Guidance for Reporting Involvement of Patients and the Public tool (GRIPP2 - short-form) (Additional file 2) [12].

### Analysis

Demographic and clinical information was taken from patient medical records and/or self-disclosure at interview. Inductive and deductive thematic analysis was guided by Braun and Clarke’s steps [13]. First, transcripts were split equally across a team of four independent researchers (AD, LD, JB, RH). Transcripts were matched with those that had conducted the interview where possible. At this point researchers familiarised themselves with the data by reading and re-reading their allocated transcripts. Next, each coded the same transcript to get a sense of reliability checking, and triangulated codes of two transcripts each for each participant group separately (staff and patients) by comparing codes and ideas across the four researchers. We then brought the patient codes to the two patient advisors in person to inform quality and reliability further. We subsequently produced an initial coding framework and used this to code the rest of the transcripts. New codes were noted during this process. All codes were transferred into the Trello software [14] and themes were grouped, consolidated, and finalised by the lead researcher (LD). The lead researcher transferred the themes and subthemes into a thematic map using Miro [15], which was then shared with the team, including the two patient advisors for their feedback and sign-off.

## Results

### Participant characteristics

Fifty-nine patients completed the quantitative phase. Twelve patients (from those who completed the quantitative phase) and twelve staff were interviewed as part of the qualitative phase. Patients had a mean age of 37.7 (SD 10.9), and staff were slightly younger (34.2 ± 12.0). On average patients had been in the hospital nearly 3 years, and staff had working at the hospital for just over 9 years (Table 1). Staff roles varied but most participants were healthcare facilitators (*n* = 6, 50%). Primary mental health diagnosis also varied but paranoid schizophrenia was the most common (*n* = 5, 42%). Diagnoses were also most commonly comorbid with other psychiatric conditions (e.g., personality disorders).

**Table 1:** Patient demographic characteristics (*n* = 24)

**Characteristics**	**Patient N (%)**	**Staff N (%)**
**Gender**		
Male	12 (100%)	6 (50%)
Female	0	3 (25%)
Prefer not to say	0	3 (25%)
**Ethnicity**		
White-British	4 (33%)	4 (33%)
Asian-British	0	2 (17%)
Black or Black British - Caribbean	2 (1%)	0
Black or Black British – African	2 (17%)	1 (1%)
Black or Black British - British	1 (1%)	0
Black or Black British – Other/Unspecified	2 (2%)	0
White - Irish	1 (1%)	1 (1%)
Prefer not to say	0	3 (25%)
**Ward level dependency**		
Assertive Rehabilitation	10 (83%)	1 (1%)
Increased support and assertive treatment	2 (17%)	2 (17%)
Admissions	0	5 (42%)
Not available	0	3 (25%)
**Primary diagnosis**		
Paranoid schizophrenia	5 (42%)	N/A
Bipolar affective disorder	2 (17%)	N/A
Emotionally unstable personality disorder	2 (17%)	N/A
Schizotypal disorder	1 (1%)	N/A
Paranoid personality disorder	1 (1%)	N/A
Mental and behavioural disorders due to multiple drug use and substance misuse	1 (1%)	N/A
**Role**		
Healthcare facilitator	N/A	6 (50%)
Ward doctor	N/A	2 (17%)
Nurse	N/A	1 (1%)
Consultant psychiatrist	N/A	1 (1%)
Senior clinical manager	N/A	1 (1%)
Clinical nurse manager	N/A	1 (1%)
**Previous use of Oxehealth before**		
Yes	N/A	2 (17%)
No	N/A	10 (83%)
	**Patient mean (SD)**	**Staff mean (SD)**
**Length of stay (days)**	1052.8 (701.2)	N/A
**Length of service at Broadmoor Hospital (years)**	N/A	9.3 (11.1)
**Length of career in mental health (years)**	N/A	11.8 (11.6)

Key: *N/A* No applicable

### Main themes

**Fig. 2:**
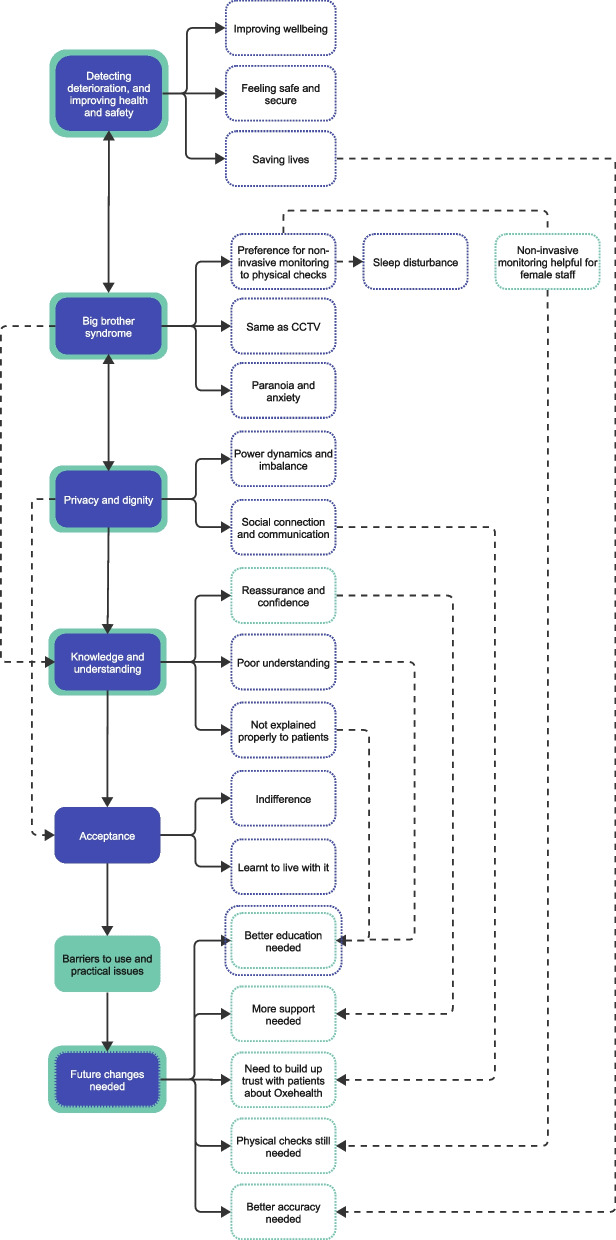
Thematic map

There were seven main themes: detecting deterioration and improving health and safety, “Big Brother Syndrome”, privacy and dignity, knowledge and understanding, acceptance, barriers to use and practice issues and future changes needed (Fig. 2).

#### Detecting deterioration and improving health and safety

Both staff and patients agreed that the main purpose of Oxehealth was to look after the patients, improve safety and ultimately save lives. Staff especially mentioned that there was a need for Oxehealth, and it was helpful to monitor the complex nature of patient’s mental and physical health (e.g., treatment-resistant patients, aggressive patients).

*“I think it’s actually really important that Broadmoor, and especially because of the complex nature of the patients and the treatment-resistant patients, how aggressive and unwell patients are and it’s a lot more difficult to take physical health, vital signs monitoring at Broadmoor, so I think it’s completely appropriate here, but it needs to be explained properly to patients and staff.” (Staff 11)*

Participants described Oxehealth as a balance between savings lives and watching the patients. For example, some patients reported feeling reassured having Oxehealth knowing that someone would intervene if their health worsened and that made them feel safer, and more relaxed. However, a few patients felt despite the intention to improve patient safety, they still did not want Oxehealth and were not happy, specifically about the invasion of privacy. Moreover, some staff and patients reported that the technology was not as accurate as it could be to detect deteriorating physical health. For example, a few patients described how a person had died in their beds despite having Oxehealth, and that Oxehealth had not detected the patient deterioration or prevented their death. Similarly, one staff member also recalled the patient death, but that Oxehealth had helped explain what had happened.

*“Well going back to that… I remember that night the alarm bell went off so I’m not sure whether that was something that Oxehealth triggered or whether that was part of the plan of Oxehealth to raise the alarm by setting off the alarm bell I’m not sure if that’s part of the plan obviously I’m not privy to what happened... but somebody died so…was it successful or did it not work but somebody died at the end of the day so if it’s there to prevent death and somebody dies then that means it failed.” (Patient 7)*

*“A patient died in their room where Oxehealth was, and I think it actually provided some timelines closer to the time when the patient is suspected to have passed away rather than…and it ruled out any assumptions that what happened what went wrong” (Staff 4, Nurse)*

*“I think he died in the area where there is a blind spot, rest in peace. So maybe if the Oxehealth could have a way of monitoring that blind spot if it was possible a bit better than it actually can or does and then that allows staff then to react, which could then save a patient’s life, react quicker maybe which could then save a patient’s life. Because I think that by the time they was made aware of him not being where he should be the time had really gone by and it was too late.” (Patient 42)*

#### “Big brother syndrome”

Patients and staff disagreed about the use and need of both invasive (physical checks every 15 min) and non-invasive (Oxehealth) patient monitoring. Most staff reported liking Oxehealth and that it was an extra safety measure that helped reassure them when they were unable to check on the patient using physical checks (e.g., patient showing aggression). However, most staff indicated physical checks should not be replaced with just Oxehealth.

*“It’s not a substitute to doing the general observation or the eyesight observations and stuff like, it’s an additional technology that you can use to help you to assure yourself that the patient is physically well” (Staff 4, Senior Clinical Manager)*

Most patients reported not having a choice in which method staff used to watch them. Most patients preferred non-invasive monitoring to physical checks despite the thought of staff watching them because the physical checks, every 15 min, were described as annoying and negatively impacted their sleep at night. For example, patients reported staff making noise and shinning lights through the windows to make sure the patients are still breathing.

*“I’ll wake up sometimes, they shine it in your eyes so you wake up, like if your watching TV and they’re disturbing you all the time shining it in” (Patient 117)*

Similarly, some female staff members reported feeling more comfortable with Oxehealth than the physical checks because they felt safer not needing to go into patient’s rooms, particularly with more aggressive and psychotic patients. In contrast, some patients were not happy with “being watched” all the time, describing it as “big brother syndrome”.

*“I still feel alright, but now I know. At first, I said to my friend, they don’t see me in my room. They can’t see me. Now I know that they can see me, sometimes I pray, and these are private things, they might have seen me pray a couple of times maybe” (Patient, 93)*

*“It’s the cameras in it, it’s all watching somebody. Like I always get changed in my bathroom and I always make sure I’m not in my room, I’m quite conscious about that.” (Patient 117)*

This invoked paranoia and anxiety from the patients, particularly when they first came into the hospital. Staff also agreed.

*“We need not to forget that our patients have a mental illness, and again, some of these paranoias and suspicions relate to being watched, you know” (Staff 4, Nurse)*

However, a few patients indicated they were not as bothered about the physical checks because staff were respectful. Other patients accepted that they were in a bad situation where connection with others was limited, specifically in seclusion. However, Oxehealth provided some patients with comfort and human connection (e.g., through the camera). Patients more than staff could not understand why staff were still doing the physical checks if they had Oxehealth. Moreover, some patients likened Oxehealth to CCTV, whereas others could see the distinction between Oxehealth and CCTV, especially staff.

#### Privacy and dignity

All participants described patients having a lack of privacy and dignity since Oxehealth technology was installed. Most patients were more bothered by the camera than Oxehealth itself (checking they are breathing), and felt it was an invasion of privacy and violation of dignity.

*“…the other day with another patient on another ward… he didn’t like it [Oxehealth] at all, he thought… his dignity was getting invaded and his privacy was getting invaded and I’ve heard a lot of other patients like when I was on [ward name] I heard other patients saying they didn’t like it in the room and it’s not fair they are being watched on cameras and that so I’ve had a lot of negative response from it.” (Patient 117)*

Some patients had adjusted their behaviour because of the camera including sleeping under the covers, getting changed in the bathroom and masturbating in the shower. One patient felt that staff were looking at him naked and felt embarrassed.

*“Er, if they see you naked, they apologise but that’s not really maintaining privacy is it.” (Patient 2)*

Most staff reported that they understood why patients felt they had no privacy, and they did their best to maintain it. For example, staff reported knocking on the door before entering, trying to reassure the patients that the camera is not recording them all the time, and giving patients the opportunity to ask questions. Most patients described feeling comfortable talking to staff if they needed to, but some often kept any issues to themselves. This two-way social interaction was apparent across all participants in some way. For example, several patients reported lack of trust between patients and staff, but staff also suggested this was something to acknowledge and a barrier to staff using Oxehealth.

*“It’s all about reassurance and information. When we build that trust up and they start to trust us a bit more, but that comes with time and then, you know, they accept more of the reassurance about it” (Staff 2, Nurse)*

A few patients reported that there was an imbalanced power dynamic that made them feel like victims, and sometimes there was a power struggle between staff and patients, but this was not just about Oxehealth. Staff views differed on whether privacy had been breached or not. For example, a healthcare facilitator felt Oxehealth did not breach ethics because staff did not abuse Oxehealth, whereas another healthcare facilitator felt there were ethical issues in watching patients. However, staff felt the benefits of Oxehealth in detecting deteriorating health outweighed the potential ethical issues.

*“There are a few points in terms of ethics in terms of the continuous recording, as well as patient privacy and also who has access to that information because I guess we are sharing information” (Staff 8, Healthcare facilitator)*

#### Knowledge and understanding

Most patients knew that Oxehealth monitored patient’s vital signs, but they all described inaccuracies about how Oxehealth works and misunderstandings about the reason for the camera and how it is used. For example, some patients felt that Oxehealth measures blood pressure *and* pulse, and others thought staff could not see them on the camera; both were inaccurate descriptions.

*“I don’t know. I think it’s something to do with lasers” (Patient 93)*

Some staff and patients described incidents where patient misunderstanding had resulted in disruption by patients including blocking the camera with wet tissues, particularly when first coming into the hospital. Notably, patients indicated that Oxehealth had not been explained to patients properly or in a consistent manner. Some patients described being told about Oxehealth during group discussion, whereas others had a one-on-one with a patient rep, and others reported being self-taught.

*“Not really, no one’s really explained to me how it works I learnt just myself by talking to some patients and that.” (Patient 117)*

Similarly, the formats of explanation also differed across the patients (e.g., leaflets, chat, and presentation). Some staff also did not know how it worked and what their role was in using Oxehealth. For example, a few staff members, especially bank staff, reported confusion over the procedural use of Oxehealth. In contrast, other staff felt confident in using Oxehealth, but Oxehealth itself made them feel confident and more assured about their own physical observations of the patient’s health and safety.

*“it’s quite reassuring for me as a junior doctor to know that people’s heart rate is not elevated, especially if you’re worried about some infection. Or that their respiratory rates particularly high, if you’re worried about breathlessness or anything like that. So, I think it’s very helpful and reassuring from that point of view.” (Staff 11, Ward Doctor)*

#### Acceptance (patient only)

Most patients reported that they had accepted that Oxehealth was here to stay and showed indifference. For example, some patients reported forgetting it was there , not paying attention to it in their rooms anymore, and not being worried about being monitored. However, they felt that nothing they said would make a difference to remove it. This view was emphasised when asked if they had a choice on the decision to keep Oxehealth or not. Some still had a problem with Oxehealth and would not want it if they had a choice. However, others could still see a place for it and felt it kept patients safe. Time seemed to be a consideration as to the reason why some patients were or were not bothered about Oxehealth with some describing that they had learned to live with it since it. Whereas those who complained about its use were more likely to have had less time in the hospital.

*“…I don’t mind it, it don’t bother me I don’t really think about it, it don’t come into my thoughts…it’s just there, part of my room.” (Patient 117)*

*“Well if I thought it was going to save people’s lives I would want it but if it was just for surveillance then I would say take it away, everyone has a right to their privacy. You know.” (Patient 22)*

#### Barriers to use and practical issues (staff only)

Staff described several practical issues to using Oxehealth. For example, most staff reported technological glitches, which included poor Wi-Fi, signal issues, not being able to view live coverage for a long period and poor readings of patient activity. Similarly, some staff indicated that there were security concerns over using the IPad to monitor Oxehealth including using it as a weapon, and patients accessing the Oxehealth data (e.g., code on the back of the IPad). Reported barriers to staff using Oxehealth were varied and more relating to personal and interpersonal factors. For example, some staff described the reliance of staff ability to use technology, not being able to understand current resources, and not being trained enough to use Oxehealth technology correctly.

*“Lack of understanding of it like me! Perhaps there might be a lack of trust in it compared to like the more familiar traditional ways of measuring the outcomes.” (Staff 2, Consultant Psychiatrist)*

#### Future changes needed

This last theme was connected to improving areas discussed in the previous themes, or sub-themes. Overall, most staff still felt physical checks were needed regardless of the Oxehealth benefits. Staff members suggested that reasons for this included having increased reassurance in knowing the patient was safe, that Oxehealth was not always accurate, and staff would not welcome swapping to Oxehealth alone. One staff member argued that Oxehealth was not a substitute but only an addition to visual checks and observations. Another staff member suggested having checks every 30 min as standard rather every 15 min. Related to this, a few staff members, and one patient mentioned that better accuracy in detecting deterioration was needed because Oxehealth did not always detect deterioration.

*“Sometimes it doesn’t always pick up readings, even when people are still. And I’ve noticed that myself when I’ve tried to check it.” (Staff 11, Ward doctor)*

Better education about Oxehealth was reported as needed for both staff and patients. Most staff described needing training in what Oxehealth does and does not do; expectations of staff, particularly bank staff, and what staff need to do to monitor patients. Subsequently, this was reported to likely improve the patients’ knowledge about Oxehealth and that it would have a positive impact on the patient’s education and acceptance. Additionally, the need for more support but from the Oxehealth company directly, was mentioned by a few staff members. Staff mentioned this would be helpful to “sort out the technology” and having more IT support on hand. One patient felt that discussing the benefits of Oxehealth with patients might help patients understand why there is a camera in their room. Another patient wanted Oxehealth in the bathroom, as they described it as the only place that could not detect deteriorating physical health and they would therefore feel safer.

*“There should be more sensors in the room, you know like, Oxehealth technology will only get better over the years but they should be one of the bathrooms” (Patient 60)*

## Discussion

### Main findings and comparison to other studies

This is the first qualitative study to explore the views of both patients and staff to evaluate the use of passive remote monitoring technology (Oxehealth) in a high-secure forensic hospital. Seven main themes were interrelated and interspersed both staff and patient views: (1) detecting deterioration and improving health and safety, (2) “big brother syndrome”, (3) privacy and dignity, (4) knowledge and understanding, (5) acceptance, (6) barriers to use and practice issues, and (7) future changes needed. Results showed there was a delicate balance between using Oxehealth to detect deterioration and improve patient safety, and maintain patients’ privacy, dignity, and human rights. For example, Oxehealth was deemed generally acceptable to staff and patients, but many patients felt it was intrusive, especially when accompanied with physical checks, and some staff indicated practical issues in its use (e.g., signal issues). Patients reiterated concerns over privacy and imbalanced power dynamic in the inpatient setting found in other studies [16, 17]. However, the safety discourse still drove the need for the ongoing use of Oxehealth, in line with other studies involving constant visual observations and electronic monitoring in inpatient settings [16].

Despite privacy concerns, and not having a choice in being observed, most patients preferred non-invasive monitoring to physical checks because the physical checks impacted their sleep and were deemed annoying. However, most staff indicated that there is a need for both Oxehealth and physical checks and that Oxehealth should not be used without physical checks because of safety concerns. This was despite the evidence showing the paradoxical impact of poor sleep being linked to subsequent aggression [3, 4] and suicide risk [18, 19] in line with other studies [20].

The lack of understanding about the technology, its infrared camera and how it works in practice was a barrier to Oxehealth’s acceptance in patients, particularly for newer patients. Going forward, several improvements would be needed to fully integrate Oxehealth successfully at Broadmoor Hospital including improved accuracy in detecting deterioration, resolving practical issues such as signal and Wi-Fi issues, more support given to both staff and patients and accurate reporting about how Oxehealth works to both new staff and patients, particularly for the first time.

### Limitations

There were several limitations. No triangulation was conducted across researchers in the final theme refinement, nor were the patient advisors able to inform on the final thematic map. This was because of restrictions in the researcher being unable to move around the high-secure hospital to talk to the patient advisors without staff support. Whilst the interview data was robust and comprehensive, patients and staff may have limited the amount and detail that they disclosed to the research assistants because of the sensitive nature of the conversations, and natural power imbalance. Patients and staff also had varied experiences in Oxehealth, and some had more experience of Oxehealth than others. As such, data could be subject to participant reporting bias. Similarly, researcher bias was possible as codes and themes could have been influenced by the research assistants (e.g., awareness of Oxehealth prior to the study), and researcher (e.g., experience in sleep research). Patients advisors with lived experience were also not able to conduct the interviews with patients themselves like in our previous studies [11, 21–23] due to security concerns, potential risk to patient advisors and disclosure of sensitive information. Half of staff participants were healthcare facilitators and therefore experiences and perspectives of other staff members (e.g., psychiatrists) were missing from the analysis and could have influenced the findings. However, this may be a potential qualitative study in the future. Finally, this study was only conducted in one high-secure hospital in the UK therefore patient and staff experiences might be difference elsewhere.

### Research and clinical implications

There are several clinical implications. There is a need for a more comprehensive and standardised way of improving patients’ understanding of Oxehealth, particularly in relation to the camera operation because of concerns over staff observing patients. Initial chats with new patients which are repeated throughout the hospital stay is required to reassure patients and increase transparency and trust between staff and patients. In addition, the development of guidance and documentation (e.g., booklet) that is co-produced with staff, researchers and patients would be a useful output for patients to refer to in addition to regular chats with staff. A better way of getting real-time support for IT issues related to Oxehealth is also needed. Despite most staff reporting on the continued need for physical checks to improve patient safety, patients could not understand why both physical checks and Oxehealth were needed. As such, there may be the potential for a feasibility study or pilot trial to examine the impact of Oxehealth on patient safety with and without the physical checks, and/or with intermittent times (e.g., 15 min, 30 min, 60 min).

## Conclusion

Passive monitoring of patient safety using Oxehealth technology was generally accepted by staff and patients within a high-secure forensic hospital but only if patient privacy and dignity was maintained. More support and education for new stakeholders is needed to uphold this view. As physical checks are still used in conjunction with Oxehealth, future research could consider a feasibility and pilot study comparing the impact of Oxehealth on patient safety with and without physical checks.

## Supplementary Information

**Additional file 1.** COREQ (COnsolidated criteria for REporting Qualitative research) Checklist.

**Additional file 2.** GRIPP2 short form.

## Data Availability

The data that support the findings of this study are available from West London NHS Trust but restrictions apply to the availability of these data, which were used under license for the current study, and so are not publicly available. Data are however available from the authors upon reasonable request and with permission of West London NHS Trust. Please contact Dr Jonathan Hafferty.

## Abbreviations

COREQ: Consolidated criteria for reporting qualitative research
GRIPP2: Guidance for Reporting Involvement of Patients and the Public tool
HCR-20: Historical Clinical Risk – 20 risk assessments
N/A: Not applicable
QUANT: Quantitative
QUAL: Qualitative
SD: Standard deviation
UK: United Kingdom

## Footnotes

1 Researchers, staff and patients know the technology as “Oxehealth” rather than the technology official name of Oxevision. Therefore, the technology will be referred to as “Oxehealth” throughout the manuscript.
