# Supplementary material for: A service evaluation of passive remote monitoring technology for patients in a high-secure forensic psychiatric hospital: a qualitative study
Source: BMC Psychiatry. 2023 Dec 14;23:946. doi: 10.1186/s12888-023-05437-w (PMC10722773; doi:10.1186/s12888-023-05437-w)
Supplement: Supplementary file 2 — Additional file 2. GRIPP2 short form. [file 12888_2023_5437_MOESM2_ESM.docx]

**Supplementary material 1: GRIPP2 short form**

| **Section and topic** | **Item** | **Reported on page No** |
| --- | --- | --- |
| 1: Aim | Report the aim of PPI in the study | 8 |
| 2: Methods | Provide a clear description of the methods used for PPI in the study | 8 |
| 3: Study results | Outcomes—Report the results of PPI in the study, including both positive and negative outcomes | N/A |
| 4: Discussion and conclusions | Outcomes—Comment on the extent to which PPI influenced the study overall. Describe positive and negative effects | 19-20 |
| 5: Reflections/critical perspective | Comment critically on the study, reflecting on the things that went well and those that did not, so others can learn from this experience | N/A (will be reported elsewhere) |
